# Diversity and distribution of fungal communities in the marine sediments of Kongsfjorden, Svalbard (High Arctic)

**DOI:** 10.1038/srep14524

**Published:** 2015-10-23

**Authors:** Tao Zhang, Neng Fei Wang, Yu Qin Zhang, Hong Yu Liu, Li Yan Yu

**Affiliations:** 1 China Pharmaceutical Culture Collection, Institute of Medicinal Biotechnology, Chinese Academy of Medical Sciences & Peking Union Medical College, Beijing 100050, P.R. China; 2 Key Lab of Marine Bioactive Substances, First Institute of Oceanography, State Oceanic Administration, Qingdao 266061, P.R. China

## Abstract

This study assessed the diversity and distribution of fungal communities in eight marine sediments of Kongsfjorden (Svalbard, High Arctic) using 454 pyrosequencing with fungal-specific primers targeting the internal transcribed spacer (ITS) region of the ribosomal rRNA gene. Sedimentary fungal communities showed high diversity with 42,219 reads belonging to 113 operational taxonomic units (OTUs). Of these OTUs, 62 belonged to the Ascomycota, 26 to Basidiomycota, 2 to Chytridiomycota, 1 to Zygomycota, 1 to Glomeromycota, and 21 to unknown fungi. The major known orders included Hypocreales and Saccharomycetales. The common fungal genera were *Pichia, Fusarium, Alternaria,* and *Malassezia*. Interestingly, most fungi occurring in these Arctic sediments may originate from the terrestrial habitats and different basins in Kongsfjorden (i.e., inner basin, central basin, and outer basin) harbor different sedimentary fungal communities. These results suggest the existence of diverse fungal communities in the Arctic marine sediments, which may serve as a useful community model for further ecological and evolutionary study of fungi in the Arctic.

Recently the Arctic marine ecosystem has received much attention because the Arctic is predicted to warm up quickly as a result of global climate change. In fact, significant impacts (e.g., impacts on species ranges, population dynamics and food web interactions) have been observed in the Arctic ecosystem^1^,^2^. It is perceived that knowledge on the structure and function of diverse microbial communities, including Bacteria, Archaea, Fungi, micro-algae, and viruses in Arctic marine ecosystems, will help to predict future changes in the Arctic^3^.

Marine fungi are a taxonomically diverse group and exist in various habitats within the marine ecosystems including sea water, corals, algae, marine sediments, and others. Obligate marine fungi grow and sporulate exclusively in the marine habitats, whereas facultative marine fungi normally occupy terrestrial or freshwater habitats but are capable of growing and probably sporulating in a marine habitat^4^. In contrast to the obligate forms, facultative marine fungi belong to terrestrial species^5^. As an example, *Aspergillus terreus* is a facultative marine fungus isolated from marine sediments, which is also distributed in terrestrial habitats and can actively grow under the deep-sea conditions^6^. Marine fungi have various ecological roles: some are major decomposers of organic matters in marine ecosystems; some are parasites, pathogens, and mutualists with other marine organisms (e.g., algae and animals)^7^; some involve in denitrification processes in marine sediments^8^,^9^. The sediments in the Arctic fjord is a unique habitat with extreme conditions such as low temperature and high sodium-ion concentration^10^. These extreme conditions provide valuable opportunities to discover extremophiles with novel genes and unique properties^11^. Understanding the fungal diversity in the marine sediments is also instrumental to study the fungal evolution because fungal divergence is believed to have been initiated in the marine habitats.

Several previous studies focused on culturable fungi within the marine sediments and reported that a high number of the isolates belonged to terrestrial species (e.g., species of *Aspergillus, Cladosporium, Penicillium*, and *Fusarium*) which have been adapted to those conditions^6^,^8^,^9^,^12^,^13^,^14^,^15^,^16^,^17^,^18^,^19^,^20^. A few studies surveyed fungi that are associated with marine sediments using molecular methods (e.g., clone libraries and 454 pyrosequencing), which enable the detection of difficult-to-culture species and rare species (e.g., species of *Malassezia* and *Pichia*)^9^,^12^,^20^,^21^,^22^,^23^,^24^,^25^,^26^. So far, the diversity and composition of marine sedimentary fungi have been reported in Arabian Sea^8^,^9^,^17^, South China Sea^16^,^26^, Bay of Bengal^18^, Sea of Japan^19^, Indian Ocean^6^,^12^,^13^,^14^,^20^,^21^,^22^, Atlantic Ocean^15^, and Pacific Ocean^23^,^24^,^25^, suggesting that these marine sediments harbor high numbers of fungal taxa. However, no reports on the fungal diversity in marine sediments from the Arctic fjord are available.

Kongsfjorden is a glacial fjord in the Arctic. It is influenced by both Atlantic and Arctic water masses (e.g., relatively warm and salty water from Atlantic Current)^27^. It also experiences the inputs from large tidal glaciers (e.g., decreased salinity, increased turbidity, and decreased light penetration)^28^. Therefore, Kongsfjorden can be a climate indicator on a local scale^29^. In this study, we used 454 pyrosequencing to investigate the fungal communities that inhabit marine sediments from Kongsfjorden (Fig. 1 and Table 1) to address the following questions: (1) what are the fungal diversity and composition in these marine sediments of the Arctic fjord? (2) whether the fungi in marine sediments of the Arctic fjord originate from the terrestrial habitats or belong to obligate marine fungi? (3) do diversity and composition of fungal communities vary among the basins and sites in Kongsfjorden?

## Results

### Sequence data

The raw data from 8 sediment samples consisted of 80,106 sequences, of which 43,916 sequences were retained after removing sequences with different tags at each end for quality filtering and denoising. After removing singletons, chimeric sequences and OTUs of nonfungal organisms or with unreliable BLAST matches (max score below 200 or aligned query sequence below 200 bp), a total of 113 fungal OTUs from 42,219 sequences were included in the final matrix. The length of 42,219 sequences ranged from 225 to 559 bp. The number of OTUs in different sediment samples ranged from 17 to 86 (Table 1).

### Fungal diversity and community structure in sediments

Based on BLASTn searches in GenBank, 113 OTUs were identified at different levels of taxonomic precision, and most of these OTUs had their best matches against GenBank accessions. Information of these 113 OTUs is presented in Table S1 and Table S2. These 113 OTUs spanned 5 phyla, 11 classes, 25 orders, 32 families, 30 genera, and 30 species. Among 113 OTUs, 62 belonged to the Ascomycota, 26 to Basidiomycota, 2 to Chytridiomycota, 1 to Zygomycota, 1 to Glomeromycota, and 21 to unknown fungi (Fig. 2).

Sequences matching with Ascomycota had high affinities with 6 known classes, with Sordariomycetes being the most abundant and diverse class. Sequences matching with Basidiomycota had high affinities with 4 known classes, with Agaricomycetes being the most abundant and diverse class (Table 2). Sequences from Ascomycota matched 12 known orders, with Hypocreales being the most abundant and the most diverse. Sequences from Basidiomycota matched 12 known orders, with Russulales being the most abundant and Sebacinales being the most diverse. The order distributions were similar for the 5 sediment samples in the outer and central basin that the abundant orders (>97% of reads in each sample) were Hypocreales and Saccharomycetales. Fungal communities in sample K6 were most diverse and had all the 25 orders that were detected in the present study (Table 2).

Of the 30 genera detected in the present study, the dominant genera were *Fusarium* (26,753 reads in 8 samples) and *Pichia* (7,241 reads in 8 samples). The common fungal species (>50 reads in more than 4 samples) were *Pichia pastoris* (7,241 reads in 8 samples)*, Fusarium proliferatum* (5,695 reads in 8 samples)*, Fusarium oxysporum* (3,321 reads in 8 samples)*, Fusarium redolens* (17,046 reads in 7 samples)*, Alternaria alternata* (95 reads in 7 samples)*, Fusarium solani* (284 reads in 5 samples), *Malassezia restricta* (131 reads in 4 samples) (Table S1).

Among 113 OTUs, 75 had the matching sequences with high similarity (≥97%). These matching sequences (≥97%) were derived from fungi found in various habitats (e.g., soil, plant tissue, water, air, etc.) within both Arctic and non-Arctic regions (e.g., USA, Norway, France, Portugal, Czech, Mexico, Japan, China, South China Sea, Pacific Ocean, Antarctica, etc.). Of these 75 OTUs, only 10 were closely related to fungi living in marine habitats, including surface sea water, subsurface sediment, and marine sponge (Table S1). The above results indicated that most of these fungal OTUs were not sediment specific and were distributed widely in marine and non-marine habitats. In addition, the other 38 fungal OTUs had the matching sequences with similarity below 97% and most of these matching sequences were derived from soils in different areas (e.g., Germany, USA, Canada, Indonesia, Japan, China, etc.), which might be unidentified fungal species.

### Dissimilarity of fungal communities among different sediment samples

The Good’s coverage estimator, Chao 1, and Shannon’s indices were used to evaluate and compare the diversity of the fungal communities among the sediment samples (Table 1). The Good’s coverage estimator ranged from 99.77 to 99.98%, indicating that 454 pyrosequencing captured the dominant phylotypes. The Chao1 index (18–86) and Shannon’s index (*H’ *= 1.51–5.02) indicated that the level of diversity varied among the 8 sediment samples.

To further understand the relationships between fungi in the Arctic marine sediments and fungi in the Arctic terrestrial habitats, a phylogenetic tree was constructed using representative sequences of fungal OTUs (Fig. 3). This result revealed that 60 fungal OTUs in Arctic sediments from Kongsfjorden were phylogenetically close to the fungi in Arctic waters from lands around Kongsfjorden. Additionally, a phylogenetic tree was constructed to elucidate the differences of fungal communities between the Arctic marine sediments and the non-Arctic marine sediments. This result demonstrated that most of sedimentary fungi in Kongsfjorden were distinct from fungi in sediments from Pacific Ocean, South China Sea, and Central Indian Basin (Figure S1).

The db-RDA analysis (Fig. 4) and hierarchical clustering analysis (Figure S2) revealed spatial distribution of the fungal communities among the different sediment samples. The db-RDA analysis showed that the fungal communities in the outer (K1 and K2) and central basin (K3, K4 and K8) were clustered, whereas the fungal communities in the inner basin (K5, K6 and K7) were separated from each other and from those in the outer and central basin. It was also shown that physicochemical factors (e.g., salinity, organic C, SiO_4_^−^, and PO_4_^3−^-P) in the outer and central basins were more similar to each other than to the ones in the inner basin (Fig. 4). MRPP test (*A *= 0.3048, *P *= 0.005 < 0.01) demonstrated significant differences existed among the fungal communities in the 3 basins (Table S3).

To gain further insight into the differences of fungal communities among the 3 basins and the 8 sampling sites, we applied Venn and network analyses of the 113 OTUs to highlight their distributions (Fig. 5). The results demonstrated that most of the fungal OTUs within the inner basin differed from those in the outer and central basin (Fig. 5a). Particularly, sampling site K6 had the highest number of OTUs, and 55 OTUs were only detected in this site (Fig. 5b).

## Discussion

The significance of fungal communities in the marine sediments of the Arctic fjord is unclear, mainly because data on the fungal species in this marine habitat are limited. This study is the first to use high-throughput sequencing in order to comprehensively analyze the fungal communities within marine sediments from Kongsfjorden (Svalbard, High Arctic), which helps to uncover fungal diversity and distribution patterns in marine sediments of the Arctic fjord.

Despite Svalbard’s geographic isolation and its extreme environmental conditions, diverse fungal communities were found in the Arctic marine sediments. Using 454 pyrosequencing, a wide range of Shannon’s indices (*H’ *= 1.51–5.02) were observed in the present study. Using traditional culture-based methods, researchers reported relatively low levels of diversity for fungal communities (*H’ *= 1.07–2.06) in the deep-sea sediments of the Central India Basin (4900 to 5390 m)^6^ and for fungal communities (*H’ *= 2.4128–3.3160) in the coastal sediments of Arabian Sea (50 to 200 m)^17^. The high sensitivity of 454 pyrosequencing enables the detection of rare species, and 454 pyrosequencing thereby provides detailed information on fungal diversity in these sediments of Arctic fjord. In addition, the depth of sediments probably has a great influence on fungal diversity.

Members of Ascomycota were more frequently identified in the Arctic marine sediments than those of Basidiomycota, whereas members of Chytridiomycota, Glomeromycota, and Zygomycota represented only a small proportion of the sedimentary fungal communities. In marine sediments of Arabian Sea (50 to 100 m), Ascomycota was the dominant phylum contributing 83%, the rest (17%) being Zygomycota^17^. These results are not in accordance with previous studies in which basidiomycete yeasts were found to be the dominant fungal forms in deep-sea environments^21^,^30^. Sequences belonging to Chytridiomycota, Glomeromycota, and Zygomycota were also recovered using molecular methods in some deep-sea habitats (e.g., sea water^30^,^31^ and sediment^9^,^20^,^30^). Additionally, a substantial portion of the fungi detected in the present study were unclassified and may be undiscovered and possibly indigenous species in Svalbard.

In our study, the most commonly identified class of Ascomycota was Sordariomycetes, followed by Saccharomycetes, Leotiomycetes, Dothideomycetes, Eurotiomycetes, and Pezizomycetes. These data are largely in agreement with a previous study of fungal communities in the deep-sea sediments of the Pacific Ocean, including Northwest Pacific Ocean (5017 to 5215 m), Central Pacific Ocean (5062 to 5145 m), and Mariana Trench area (6986 m)^23^. The most abundant class in sediments of the Pacific Ocean was Sordariomycetes, followed by Dothideomycetes, Saccharomycetes, Eurotiomycetes, and Leotiomycetes^23^. However, phylogenetic analysis indicates that most of the 113 fungal OTUs in Kongsfjorden are distinct from those in sediments from Pacific Ocean, South China Sea and Central Indian Basin, suggesting the fungal communities in Arctic sediments may be strongly influenced by environmental conditions in the Arctic fjord.

Among the 30 fungal genera found in this study, 14 genera have been previously observed in the marine sediments from different oceans, including *Acremonium*^14^,^15^,^16^*, Aspergillus*^12^,^13^,^14^,^15^,^16^,^17^,^18^,^19^,^20^,^21^,^22^,^23^*, Cladosporium*^16^,^17^,^18^*, Eurotium*^12^,^15^*, Fusarium*^6^,^23^,^24^*, Gloeotinia*^23^, *Malassezia*^12^,^21^*, Mortierella*^17^,^20^*, Paecilomyces*^17^*, Pichia*^21^,^26^*, Phoma*^18^,^20^*, Rhodotorula*^9^,^12^*, Trametes*^12^,^23^, and *Trichosporon*^20^,^21^,^22^,^23^,^24^,^25^. One of the most abundant OTUs is affiliated to *Fusarium*, which was observed in all 8 Arctic sediment samples. *Fusarium* species have been reported in a number of marine areas, including deep subsurface sediments (down to 346 metres below seafloor) in basin near New Zealand^24^, sediments in the Central Indian Basin (at the depth of 4900 to 5390 m)^6^, and deep-sea sediments in the Pacific Oceans (5017 to 6986 m)^23^. Representatives of genus *Pichia*, which were common in all 8 sediment samples, have the ability to colonize marine environments, including methane hydrate bearing deep-sea sediments in South China (~3,000 m)^26^ and deep-sea sediments in the Central India Basin (~5,000 m)^21^. Interestingly, some other genera have been found in terrestrial habitats of the Arctic. For example, the genus *Mortierella* was detected in the Arctic soils^32^; members of *Sebacina* were observed in plant roots from the Arctic and alpine regions^33^. The genera *Mrakia, Malassezia, Rhodotorula,* and *Trichosporon* are extremophilic yeasts that were frequently found in the Arctic, Antarctic and alpine habitats^34^.

Marine sediment represents one environmental niche in which two distinct groups can be found, including the fungi that originate from the terrestrial environment and the fungi that live and propagate exclusively in the marine habitats^4^. In this study, a large proportion of matching fungal sequences (≥97% similarity) are from terrestrial habitats and most fungal OTUs belong to known terrestrial fungal genera, such as *Fusarium, Aspergillus, Phoma, Cladosporium, Mortierella, Sebacina*, and *Alternaria*. In addition, most of the fungi in sediments are closely related to fungi in waters from lands around Kongsfjorden. These results suggest that most of the fungal taxa are derived from terrestrial habitats. In terms of the route of transport, spores and fungal hyphae may arrive in seawater of Kongsfjorden through terrestrial runoff waters from melting ice and get deposited in the bed of Kongsfjorden by sedimentation together with other particles.

The only differentiation between marine and terrestrial fungi is that marine fungi have been adapted to grow and tolerate marine environment^35^. Pawar & Thirumalachar^35^ studied the growth of marine and terrestrial isolates of the same species, and found that most of the marine isolates grew better on seawater agar, whereas the terrestrial isolates of the same species grew better on non-seawater agar. In a previous study, spores of a deep-sea isolate *Aspergillus terreus* were found to grow under extreme conditions in deep-sea environment^6^. The fungi detected in this study may have adapted to Arctic marine sediment conditions, including low temperatures and high sodium-ion concentration. Indeed, quite a few fungi developed effective strategies to tolerate cold stress, such as increasing in unsaturated membrane lipids (e.g., *Cladosporium cladosporioides, Mrakia frigida*), synthesis of antifreeze and cold shock proteins (e.g., *Mrakia* sp., *Rhodotorula* spp.), and cold-induced accumulation of trehalose and glycerol (e.g., *Saccharomyces cerevisiae*)^34^,^36^. Additionally, some fungal species could produce stress proteins to adapt high sodium-ion concentration. For example, the ribosomal protein L44 (RPL44) screened from *Aspergillus* species was associated with salt resistance^37^.

The ecological roles of these fungi in the Arctic sediments warrant further investigation. In the previous studies, a few fungal species were found to play functional roles in the marine habitats. For example, *Fusarium oxysprum* and *Aspergillus oryzae* could grow under oxygen deficient conditions and participated in denitrification processes^8^,^9^; *Fusarium oxysporum* and *Fusarium solani* were pathogenic to prawns^38^,^39^.

A distinct spatial distribution of fungal communities in Kongsfjorden was observed in the present study. Notably, sampling site K6 had the highest number of fungal OTUs and 15 genera were only detected in this site, including *Amanita, Boletellus, Chloridium, Cladophialophora, Curvibasidium, Engyodontium, Mortierella, Nolanea, Paecilomyces, Pestalotiopsis, Piloderma, Sebacina, Trametes, Trichosporon*, and *Venturia*. The distinct spatial distribution of fauna in Kongsforden was observed by Włodarska-Kowalczuk & Pearson (2004)^40^, who found that fauna assemblages within sediments differed between the inner basin and the outer basin.

What lead to this distinct spatial distribution of fungal communities? We believe that the glacial activity in the inner fjord has great impact on these marine sediments from the 8 sampling sites, especially K5, K6 and K7 in the inner basin. The glacial activity creates environmental gradients in salinity, temperature, sedimentation rates and bottom sediment composition^41^. The mineral materials, carried by glacier meltwaters, move into the inner fjord every season and the sediments become stabilized towards the central basin^42^. In the summer, a large amount of the runoff waters with soils are input into adjacent fjord waters, which may bring a large number of terrestrial fungal taxa into Kongsfjorden. Because spores and fungal hyphae are usually deposited in the bed of Kongsfjorden by sedimentation, the difference in sediment accumulation rate (i.e., 20000 g m^–2^ yr^–1^ in the inner fjord, 1800 to 3800 g m^–2^ yr^–1^ in the central fjord, and 200 g m^–2^ yr^–1^in the outer fjord^41^) may give rise to differential fungal composition and diversity in sediments from site K1 to K8 (at depths ranging from 39 to 250 m). The environmental conditions in different basins of Kongsfjorden may also influence the sedimentary fungal communities. In contrast to the inner basin of the fjord (e.g., low salinity, low contents of organic C, SiO_4_^−^, and PO_4_^3−^-P), the outer basin (depths of about 250 m) and the central basin (depths of about 100 m) have similar physicochemical conditions (e.g., salinity, organic C, SiO_4_^−^, and PO_4_^3−^-P) and are less affected by runoff waters from lands. Therefore, these two basins harbor relatively similar fungal communities in the Arctic marine sediments.

In summary, the above results indicate that marine sediments in the Arctic fjord are an important niche that harbors many fungal taxa, most of which possibly originate from the terrestrial environment. However, the ecological roles of these fungi and their adaptive mechanism remain poorly investigated. It is also unclear if these fungi are actively growing in the sediments or being dormant propagules (spores) that are washed into the sediments. A combination of different technologies including traditional culture-based method, metagenomics, metatranscriptomics, and metaproteomics may help to answer these pending questions.

## Methods

### Study sites and sample collection

The study area is located in Kongsfjorden, which is an open, 26 km long fjord situated on north-western coast of Spitsbergen, the main island of Svalbard archipelago (79°N, 12°E). The inner basin of the fjord (glacial bays) is on average 40–60 m deep and is well separated from the main fjord by a chain of islands. The outer basin has average depths of 200–300 m. The inner basin has an active glacier, Kongsbreen^43^.

**Figure 1:**
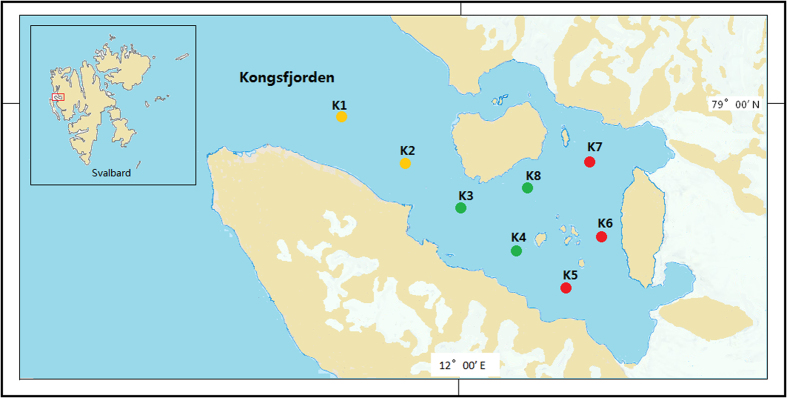
Map of Kongsfjorden in Svalbard (High Arctic) showing the sites where sediment samples were collected for this study (created using drawing tool software in Window 8.0). Three sites in the inner basin are indicated by red dots, three sites in the central basin indicated by green dots, and two sites in the outer basin indicated by yellow dots.

**Figure 2:**
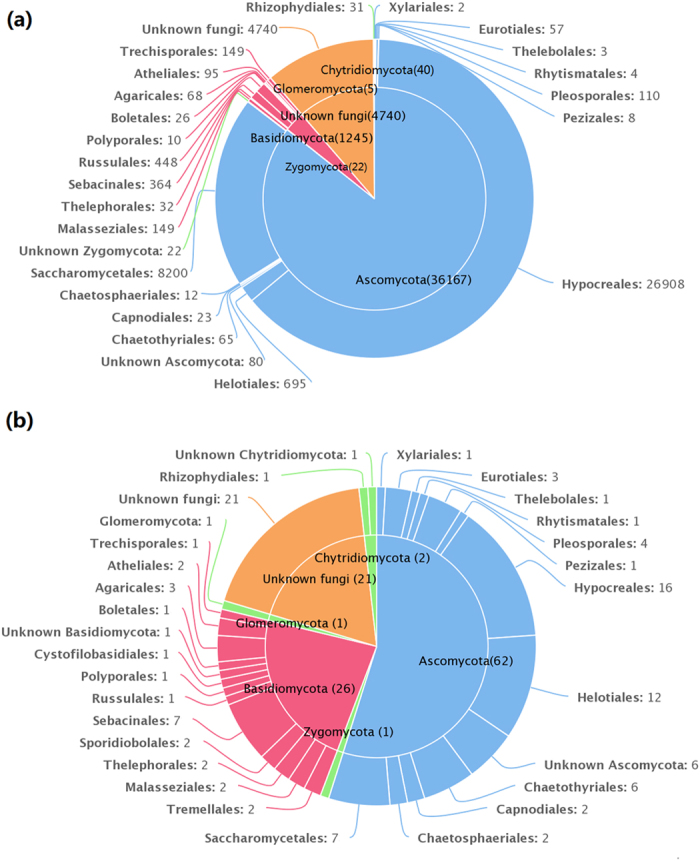
(**a**) The taxonomic distribution of 42,219 sequences at the order level. (**b**) The taxonomic distribution of 113 OTUs at the order level.

**Figure 3:**
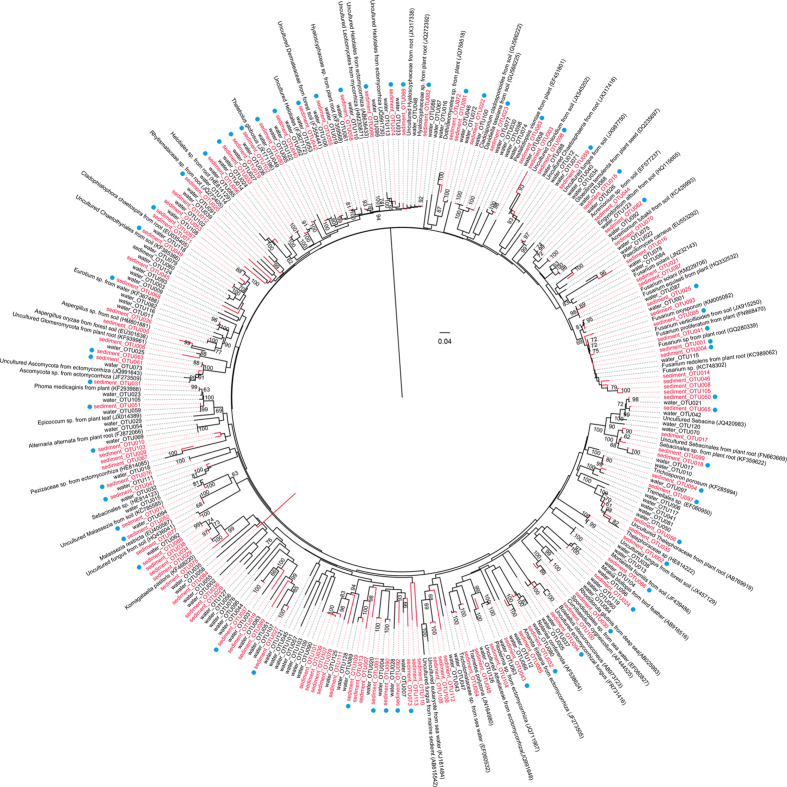
A phylogenetic tree showing the relationship between the sedimentary fungi in Kongsfjorden (113 fungal OTUs in red) and fungi in Arctic waters from lands around Kongsfjorden (128 fungal OTUs). The phylogenetic tree was constructed based on the ITS sequence using the Neighbor-Joining method with the maximum composite likelihood substitution model. Bootstrap values for 1,000 replicates are given in the branch nodes. The OTUs indicated by a blue dot are phylogeneticlly close to fungal OTUs from Arctic waters.

**Figure 4:**
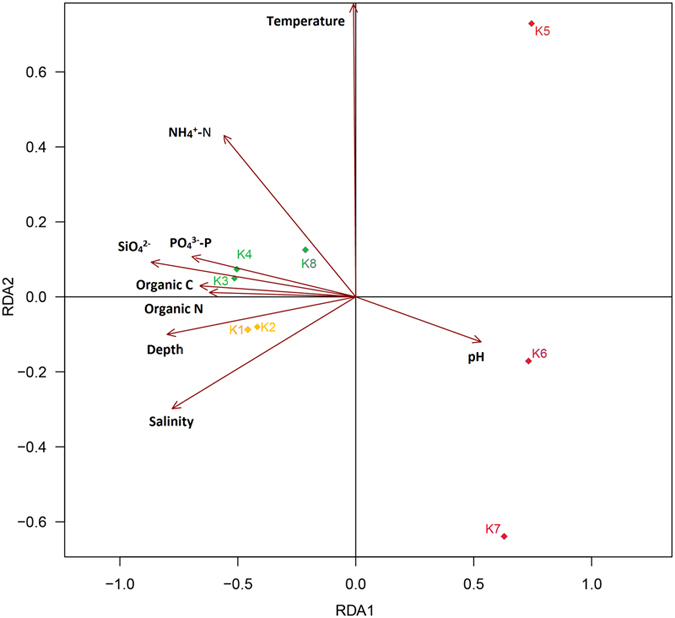
Distance based redundancy analysis (db-RDA) of square-root-transformed Bray-Curtis distances on abundance data for the 113 fungal OTUs. The different colors/symbols represent the 8 sediment samples. Three samples in the inner basin are indicated by red diamonds, three samples in the central basin indicated by green diamonds, and two samples in the outer basin indicated by yellow diamonds. Rectangles represent physicochemical factors.

**Figure 5:**
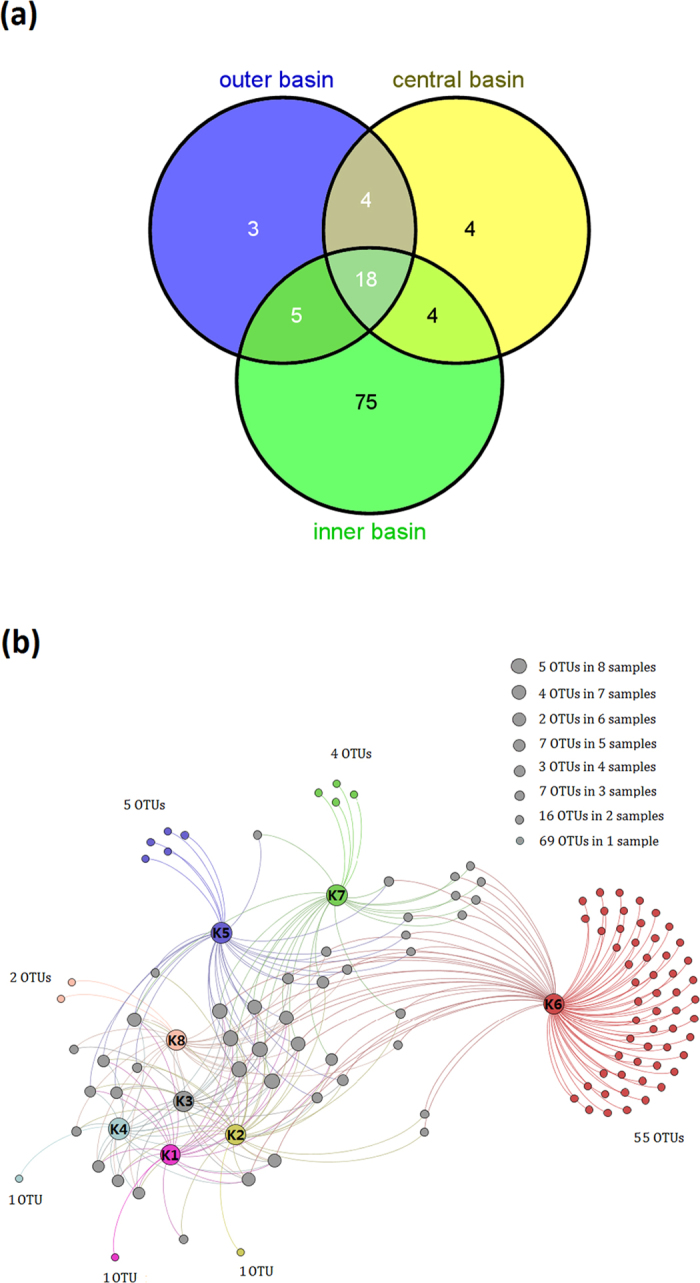
(**a**) A Venn diagram showing the degree of overlap of fungal OTUs among the 3 different basins. (**b**) A Gephi network diagram illustrating the 113 fungal OTUs and highlighting the number of shared OTUs among the 8 sediment samples.

Sampling was performed during China’s Arctic expedition in July 2013. Surface sediments were collected using a grab sampler operated from a boat and then directly placed into TWIRL’EM sterile sampling bags (Labplas Inc., Ste-Julie, QC, Canada). Two sampling sites (K1 to K2) were in the outer basin, three sampling sites (K3, K4, K8) in the central basin, and three sampling sites (K5 to K7) in the inner basin (Fig. 1). Samples were frozen at −20 °C in the Yellow River Station (China) and transported to our home laboratory by flight. Samples were then stored at −80 °C until nucleic acids were extracted.

### Physical and chemical analysis of sediments

A total of eight physicochemical properties of sediments were analyzed, including temperature, salinity, pH, organic carbon, organic nitrogen, ammonium nitrogen (NH_4_^+^-N), silicate (SiO_4_^2−^), and phosphate phosphorus (PO_4_^3−^-P). Temperature and salinity were measured by a CTD (SBE 19 plus CTD, Sea-Bird Electronics, Bellevue, Washington, USA) at each sampling site. In the laboratory, pH was measured by adding 10 ml of distilled water to 4 g of sediments and recording pH using a pH electrode (PHS-3C, Shanghai REX Instrument Factory, Shanghai, China). Analysis of organic carbon and organic nitrogen was performed using an Elemental Analyzer (EA3000, Euro Vector SpA, Milan, Italy). The other chemical properties were analyzed using a High Performance Microflow Analyzer (QuAAtro, SEAL Analytical GmbH, Norderstedt, Germany).

### DNA extraction

An aliquot of 0.25 g (wet weight) of surface sediments (three replicates for each sample) was placed in a tube containing stainless glass grinding beads. The SuperFastPrep-1^TM^ Instrument (MP Biomedicals, Santa Ana, CA, USA) was used to homogenize the sediment samples for 1 min. After grinding process, DNA was extracted using a PowerSoil DNA Isolation Kit (MO BIO Laboratories, San Diego, CA, USA) according to the manufacturer’s instructions. The resulting DNA extracts were used for the subsequent PCR and sequencing experiments.

### 454 pyrosequencing

The fungal internal transcribed spacer (ITS) region of nuclear ribosomal DNA sequences was amplified using the primers ITS1F and ITS4^44^. The PCR amplification was performed using amplicon fusion primers as 5′-A-x-ITS1F-3′ and 5′-B-ITS4-3′, where A and B represent the pyrosequencing adaptors and x represents an 8 bp-tag for the sample identification (Table S4). The 20 μl reaction mixture contained the template DNA (10 ng of Template DNA), 4 μl of 5×buffer, 2 μl of 2.5 nM dNTP, 0.8 μl of Fastpfu Polymerase, 2 μM of each primer and ddH_2_O. PCR started with an initial denaturation at 95 °C for 2 min, followed by 30 cycles of denaturation at 95 °C for 30 s, annealing at 55 °C for 30 s, and extension at 72 °C for 30 s, and a final extension at 72 °C for 5 min. Three PCR replicates for each sample were pooled prior to purifying and sequencing. After purification using the AxyPrep DNA Gel Extraction Kit (Axygen Biosciences, Corning, NY, USA) and quantification using QuantiFluor-ST (Promega Corporation, Madison, WI, USA), the PCR amplicons from each sample were mixed in equimolar amounts and then pyrosequenced using the 454 GS FLX + Platform (Roche Applied Science, Indianapolis, IN, USA) at Majorbio Bio-Pharm Technology Co., Ltd., Shanghai, China. The raw sequences were deposited in the NCBI sequencing read archive (SRA) under Accession No. SRP053984.

### Pyrosequencing data processing

Raw sequence data generated from pyrosequencing were processed using QIIME 1.8.0 software^45^. In brief, the sequence libraries were split and denoised to avoid diversity overestimation caused by sequencing errors, including sequences with average quality score <20 over a 50 bp sliding window, sequences shorter than 200 bp, sequences with homopolymers longer than six nucleotides, and sequences containing ambiguous base calls or incorrect primer sequences. Operational Taxonomic Units (OTUs) were clustered with 97% similarity cutoff using UPARSE^46^ and chimeric sequences were identified and removed using UCHIME^47^. The most abundant sequence for each OTU was chosen as a representative sequence. These OTUs were used to calculate alpha-diversity indices (i.e., Chao1, Good’s coverage estimator, and Shannon) and beta-diversity metrics using QIIME 1.8.0 software^45^.

### Statistical analyses

Sequences representing the OTUs were subjected to BLASTn search in GenBank (http://www.ncbi.nlm.nih.gov/genbank/) in order to determine their taxonomic affiliation. The following criteria were used to interpret the sequences. For sequence identities ≥97%, the genus and species were accepted, for sequence identities between 95% and 97%, only the genus was accepted, and for sequence identities <95%, OTUs were labeled at the order, family or phylum name or as ‘unassigned’. A phylogenetic tree was constructed to illustrate the relationships between the fungi in Arctic sediments from Kongsfjorden and those in Arctic waters from lands around Kongsfjorden (NCBI SRA Accession No. SRP049681) using MEGA v. 6.0 and the Neighbor-Joining algorithm, with bootstrap values calculated from 1,000 replicate runs. The ITS sequences were aligned using a multiple sequence alignment program MAFFT version 7 (http://mafft.cbrc.jp/alignment/server/). The relevance of environmental variables in explaining the distribution patterns of fungal communities in the eight sediments was analyzed by distance based redundancy analysis (db-RDA) using R 3.1.1 statistical software. A Venn diagram of shared and unique OTUs among the 3 basins was generated using Venny 2.0 (http://bioinfogp.cnb.csic.es/tools/venny/index. html). Network analysis was performed to visualize the distribution of 113 OTUs among the 8 sampling sites using Gephi 0.8.2 software^48^.

## Additional Information

**How to cite this article**: Zhang, T. *et al.* Diversity and distribution of fungal communities in the marine sediments of Kongsfjorden, Svalbard (High Arctic). *Sci. Rep.* **5**, 14524; doi: 10.1038/srep14524 (2015).

## Supplementary Material

Supplementary Information

## Figures and Tables

**Table 1:** Description of the 8 sediment samples investigated in the present study.

**Sampling code**	**Sampling date**	**Coordination**	**Depth (m)**	**Temperature (°C)**	**Salinity (psu)**	**pH**	**Organic C (mg/g)**	**Organic N (mg/g)**	**NH**_**4**_^**+**^**-N (μg/g)**	**SiO**_**4**_^**2**−^ **(μg/g)**	**PO**_**4**_^3**—**^**P (μg/g)**	valid*	**trimmed**#	**OTU**§	**Chao1**	**Good’s coverage estimator (%)**	**Shannon (*****H’***)
K1	2013.7.5	78°59′17.40′′N; 11°39′36.00′′E	248.85	1.678	35.04	8.24	8.33	1.922	22.23	44.04	2.11	16620	5527	21	21	99.96	2.39
K2	2013.7.5	78°58′00.00′′N; 11°49′41.40′′E	199.76	2.378	35.02	8.25	3.02	0.695	15.95	42.24	2.12	12229	4507	29	34	99.86	2.43
K3	2013.7.7	78°56′42.60′′N; 11°59′07.80′′E	249.49	1.856	35.03	8.32	5.46	1.109	48.55	70.69	7.56	7867	4746	17	18	99.93	1.75
K4	2013.7.7	78°55′28.20′′N; 12°08′45.00′′E	107.53	2.409	34.82	8.56	2.69	0.548	23.32	41.60	3.35	6562	4397	18	18	99.95	1.51
K5	2013.7.7	78°54′17.40′′N; 12°17′48.00′′E	41.30	2.990	34.56	8.48	1.86	0.376	23.86	16.86	0.69	3106	2228	30	32	99.77	1.56
K6	2013.7.9	78°56′00.00′′N; 12°22′20.40′′E	39.49	2.652	34.42	8.52	0.83	0.116	4.22	9.95	0.31	9595	2489	86	86	99.83	5.02
K7	2013.7.9	78°57′43.80′′N; 12°20′54.00′′E	62.36	0.310	34.86	8.63	1.19	0.206	5.27	13.25	0.32	11196	8107	24	24	99.98	2.51
K8	2013.7.9	78°57′13.80′′N; 12°10′00.00′′E	79.75	2.196	34.74	8.60	1.96	0.196	14.11	28.28	1.19	12931	10218	25	25	99.98	1.53

^*^Number of valid sequences.

^#^Number of trimmed sequences.

^§^Number of OTUs.

**Table 2:** Information on the classes and orders with which the OTUs and reads presented in the 8 sediment samples.

	**% of reads**	**% of OTUs**	**Outer basin**		**Central basin**			**Inner basin**		
			**K1**	**K2**	**K3**	**K4**	**K8**	**K5**	**K6**	**K7**
**Ascomycota**	**85.67**	**54.87**	**99.54**	**97.86**	**99.95**	**99.89**	**99.51**	**3.45**	**35.06**	**74.01**
Dothideomycetes	0.31	5.31	0.11	0.24	0.06	−	0.09	1.17	2.29	0.26
Capnodiales	0.05	1.77	−	0.02	−	−	0.02	0.18	0.64	−
Pleosporales	0.26	3.54	0.11	0.22	0.06	−	0.07	0.99	1.65	0.26
Eurotiomycetes	0.29	7.96	0.65	0.11	0.04	0.07	−	0.22	2.81	0.01
Chaetothyriales	0.15	5.31	−	−	−	−	−	−	2.61	−
Eurotiales	0.14	2.65	0.65	0.11	0.04	0.07	−	0.22	0.20	0.01
Leotiomycetes	1.67	12.39	−	0.09	−	−	−	−	21.37	2.04
Helotiales	1.65	10.62	−	0.09	−	−	−	−	21.17	2.02
Rhytismatales	0.01	0.88	−	−	−	−	−	−	0.16	−
Thelebolales	0.01	0.88	−	−	−	−	−	−	0.04	0.02
Top lattice	0.02	0.88	−	−	−	−	−	−	0.32	−
Pezizales	0.02	0.88	−	−	−	−	−	−	0.32	−
Saccharomycetes	19.42	6.19	21.60	20.10	4.21	0.41	0.21	0.85	2.25	71.37
Saccharomycetales	19.42	6.19	21.60	20.10	4.21	0.41	0.21	0.85	2.25	71.37
Sordariomycetes	63.77	16.81	77.18	77.32	95.64	99.34	99.21	1.21	2.93	0.33
Chaetosphaeriales	0.03	1.77	−	−	−	−	−	−	0.48	−
Hypocreales	63.73	14.16	77.18	77.32	95.64	99.34	99.21	1.21	2.37	0.33
Xylariales	0.01	0.88	−	−	−	−	−	−	0.08	−
Unknown Ascomycota	0.19	5.31	−	−	−	0.07	−	−	3.09	−
**Basidiomycota**	**2.94**	**23.01**	−	**0.16**	**0.04**	−	**0.05**	**0.58**	**47.77**	**0.36**
Agaricomycetes	2.53	15.93	−	−	0.02	−	0.01	0.27	42.14	0.12
Agaricales	0.16	2.65	−	−	−	−	−	−	2.73	−
Atheliales	0.23	1.77	−	−	−	−	−	−	3.42	0.12
Boletales	0.06	0.88	−	−	−	−	−	−	1.04	−
Polyporales	0.02	0.88	−	−	−	−	−	−	0.40	−
Russulales	1.06	0.88	−	−	0.02	−	0.01	0.27	17.68	−
Sebacinales	0.86	6.19	−	−	−	−	−	−	14.62	−
Thelephorales	0.08	1.77	−	−	−	−	−	−	1.29	−
Trechisporales	0.06	0.88	−	−	−	−	−	−	0.96	−
Exobasidiomycetes	0.35	1.77	−	0.16	0.02	−	−	0.22	4.86	0.19
Malasseziales	0.35	1.77	−	0.16	0.02	−	−	0.22	4.86	0.19
Microbotryomycetes	0.02	1.77	−	−	−	−	−	0.09	0.28	−
Sporidiobolales	0.02	1.77	−	−	−	−	−	0.09	0.28	−
Tremellomycetes	0.02	2.65	−	−	−	−	0.04	−	1.16	0.05
Cystofilobasidiales	0.01	0.88	−	−	−	−	0.04	−	0.08	−
Tremellales	0.01	1.77	−	−	−	−	−	−	0.08	0.05
Unknown Basidiomycota	0.02	0.88	−	−	−	−	−	−	0.32	−
**Chytridiomycota**	**0.09**	**1.76**	**0.05**	**0.02**	−	−	−	**0.22**	**1.25**	−
Chytridiomycetes	0.07	0.88	−	−	−	−	−	−	1.25	−
Rhizophydiales	0.07	0.88	−	−	−	−	−	−	1.25	−
Unknown Chytridiomycota	0.02	0.88	0.05	0.02	−	−	−	0.22	−	−
**Glomeromycota**	**0.01**	**0.88**	−	−	−	−	−	−	**0.20**	−
**Zygomycota**	**0.05**	**0.88**	−	−	−	−	−	−	**0.88**	−
**Unknown fungi**	**11.23**	**18.58**	**0.39**	**1.95**	−	**0.11**	**0.45**	**95.74**	**14.83**	**25.62**

The second and third columns indicate the percentage of total reads and total number of OTUs across the sediment samples, respectively. The last 8 columns provide a taxonomic overview of the fungal communities found in each of the 8 sediment samples, which are represented as the percentage of reads.
