# Supplementary material for: Diversity and distribution of fungal communities in the marine sediments of Kongsfjorden, Svalbard (High Arctic)
Source: Sci Rep. 2015 Oct 23;5:14524. doi: 10.1038/srep14524 (PMC4615975; doi:10.1038/srep14524)
Supplement: Supplementary Information [file srep14524-s1.pdf]

Supplementary Material

Diversity and distribution of fungal communities in the marine sediments of Kongsfjorden, Svalbard (High Arctic)

Tao Zhang[1], Neng Fei Wang[2], Yu-Qin Zhang[1], Hong-Yu Liu[1] & Li-Yan Yu[1*]

1. China Pharmaceutical Culture Collection, Institute of Medicinal Biotechnology, Chinese Academy of Medical Sciences & Peking Union Medical College, Beijing 100050, PR China

2. Key Lab of Marine Bioactive Substances, First Institute of Oceanography, State Oceanic Administration, Qingdao 266061, PR China

* Corresponding Author E-mail: yly@cpcc.ac.cn; Tel/fax:+86 10 63187118

Table S1. Information on the OTUs found in the 8 sediment samples, including their frequency, number of reads and BLASTN top hits with accession numbers in GenBank.

OTU ID	Freq.	Reads	phylum	Order	Family	Indendification	Close GenBank match / Accession	Similarity	Originally reported habitat
1	7	17046	Ascomycota	Hypocreales	Nectriaceae	*Fusarium redolens**	Uncultured fungus clone FN397215	550/553(99%)	Soil in France
							Fusarium redolens KC989062	546/553(99%)	Roots in Sweden
2	1	24	Basidiomycota	Trechisporales	Unassigned	Trechisporales	Uncultured Trechisporales clone JF691126	324/356(91%)	Plant roots in Reunioin
3	1	3	Ascomycota	Chaetosphaeriales	Chaetosphaeriaceae	*Chloridium***	Uncultured *Chloridium* clone JX545202	224/225(99%)	Soil in USA
4	8	5695	Ascomycota	Hypocreales	Nectriaceae	*Fusarium proliferatum**	*Fusarium proliferatum* FN868470	392/402(98%)	Spanish stands of *Pinus halepensis*
5	3	7	Ascomycota	Eurotiales	Aspergillaceae	*Aspergillus oryzae**	*Aspergillus oryzae* EU301638	270/271(99%)	Forest soilin China
6	1	5	Glomeromycota	Unassigned	Unassigned	Glomeromycota	Uncultured Glomeromycota clone KF939961	270/295(92%)	Plant root in Tibet
7	1	2	Unassigned	Unassigned	Unassigned	Fungus	Uncultured fungus clone KF800416	305/369(83%)	Soil
8	6	80	Ascomycota	Hypocreales	Nectriaceae	*Fusarium***	Uncultured *Fusarium* clone GQ280339	240/245(98%)	Plant roots
9	1	3	Ascomycota	Saccharomycetales	Metschnikowiaceae	Metschnikowiaceae	*Metschnikowia cibodasensis* AB236922	357/390(92%)	Plant flowers in Indonesia
10	7	95	Ascomycota	Pleosporales	Pleosporaceae	*Alternaria alternata**	*Alternaria alternata* FJ872066	521/521(100%)	Plant roots of *Pinus mugo*
11	2	18	Basidiomycota	Malasseziales	Malasseziaceae	*Malassezia***	Uncultured eukaryote clone GU941443	485/486(99%)	South China Sea
							Uncultured *Malassezia* clone KC785585	486/491(99%)	Soil in Antarctica
12	1	2	Ascomycota	Unassigned	Unassigned	Ascomycota	Uncultured fungus clone JX043002	345/368(94%)	Ectomycorrhiza in USA
							Uncultured Ascomycota clone EU490097	339/393(86%)	Savanna soil
13	1	3	Unassigned	Unassigned	Unassigned	Fungus	Uncultured soil fungus clone JX489834	299/374(80%)	Soil in China
14	5	39	Ascomycota	Hypocreales	Nectriaceae	*Fusarium***	Uncultured *Fusarium* clone GQ280339	427/434(98%)	Plant roots
15	2	37	Ascomycota	Helotiales	Unassigned	*Gloeotinia temulenta**	Uncultured eukaryote clone KJ182739	533/534(99%)	Surface sea water
							Gloeotinia temulenta DQ235697	532/534(99%)	Plant seed
16	1	3	Ascomycota	Hypocreales	Clavicipitaceae	*Paecilomyces carneus**	*Paecilomyces* sp. GQ241283	243/249(98%)	Wastes
							Paecilomyces carneus EU553292	214/219(98%)	Unreported
17	1	6	Basidiomycota	Sebacinales	Unassigned	Sebacinales	Uncultured Sebacinales clone FN663669	404/411(98%)	Plant roots
18	1	16	Basidiomycota	Sebacinales	Unassigned	Sebacinales	Uncultured Sebacinales clone FJ475786	366/392(93%)	Forest soil in Sweden

OTU ID	Freq.	Reads	phylum	Order	Family	Indendification	Close GenBank match / Accession	Similarity	Originally reported habitat
19	1	8	Basidiomycota	Unassigned	Unassigned	Basidiomycota	Uncultured Basidiomycota clone HM240234	463/492(94%)	Grassland soil in USA
20	1	3	Ascomycota	Unassigned	Unassigned	Dothideomycetes	Uncultured soil fungus clone EU490120	471/504(93%)	Savanna soil
							Dothideomycetes sp. AB847071	418/448(93%)	Plant roots in Japan
21	2	7	Ascomycota	Hypocreales	Nectriaceae	*Fusarium solani**	*Fusarium solani* KM229706	363/374(97%)	Unreported
22	2	10	Ascomycota	Capnodiales	Davidiellaceae	*Cladosporium cladosporioides**	*Cladosporium* sp. HG008746	467/468(99%)	Air sample in France
							Cladosporium cladosporioides GU566222	467/468(99%)	Rhizosphere in Czech
23	3	13	Ascomycota	Capnodiales	Davidiellaceae	*Davidiella tassiana**	*Davidiella tassiana* GU566225	558/559(99%)	Rhizosphere
24	2	6	Basidiomycota	Cystofilobasidiales	Cystofilobasidiaceae	*Mrakia blollopis**	*Mrakia blollopis* AB916516	406/410(99%)	Arctic bird feather in Norway
25	4	17	Ascomycota	Hypocreales	Nectriaceae	*Fusarium equiseti**	*Fusarium* sp. EU330614	544/544(100%)	Mangrove plant in China
							Fusarium equiseti HQ332532	530/530(100%)	Plant
26	4	9	Ascomycota	Eurotiales	Aspergillaceae	*Aspergillus tubingensis**	*Aspergillus* sp. HM801881	455/456(99%)	Rock phosphate mine landfills
							Aspergillus tubingensis HQ728255	449/449(100%)	Tea
27	2	12	Unassinged	Unassinged	Unassigned	Fungus	Uncultured fungus clone AM260830	377/449(84%)	Peat in United Kingdom
28	1	5	Ascomycota	Chaetothyriales	Unassigned	Chaetothyriales	Uncultured Chaetothyriales clone HM136629	247/266(93%)	Rhizosphere soil
29	7	217	Ascomycota	Saccharomycetales	Phaffomycetaceae	*Pichia pastoris**	*Pichia pastoris* FR839630	368/379(97%)	Unassigned
30	1	2	Basidiomycota	Sporidiobolales	Unassinged	*Rhodotorula glutinis**	*Rhodotorula glutinis* AB025993	508/513(99%)	Deap sea environments around the northwest Pacific Ocean
31	3	6	Ascomycota	Pleosporales	Didymellaceae	*Phoma medicaginis**	*Phoma medicaginis* KF293988	521/521(100%)	Plant stem in China
32	1	61	Basidiomycota	Agaricales	Entolomataceae	*Nolanea conferenda**	*Nolanea* sp. JQ272420	451/458(98%)	USA
							Nolanea conferenda AF538624	408/413(99%)	Unreported
33	1	21	Ascomycota	Helotiales	Helotiaceae	Helotiaceae	Uncultured Helotiaceae clone FJ440900	447/494(90%)	Plant roots
34	8	3147	Ascomycota	Saccharomycetales	Phaffomycetaceae	*Pichia pastoris**	*Pichia pastoris* FR839631	384/387(99%)	Unreported
35	1	15	Basidiomycota	Thelephorales	Thelephoraceae	Thelephoraceae	Thelephoraceae sp. HE814222	279/281(99%)	Root tips in China
36	1	3	Basidiomycota	Sebacinales	Sebacinaceae	*Sebacina***	Uncultured *Sebacina* clone JQ318631	220/222(99%)	Plant

OTU ID	Freq.	Reads	phylum	Order	Family	Indendification	Close GenBank match / Accession	Similarity	Originally reported habitat
37	1	4	Unassigned	Unassigned	Unassigned	Fungus	Uncultured fungus clone EF619893	395/424(93%)	Forest soil in USA
38	8	1645	Ascomycota	Saccharomycetales	Phafomycetaceae	*Pichia pastoris**	*Pichia pastoris* FN392325	393/400(98%)	Unreported
39	5	1810	Unassigned	Unassigned	Unassigned	Fungus	Uncultured soil fungus clone EU480016	305/386(79%)	Rhizosphere soil in USA
40	1	64	Ascomycota	Helotiales	Unassigned	Helotiales	Uncultured Helotiales clone FJ827172	348/350(99%)	Ectomycorrhiza in China
41	3	6	Ascomycota	Hypocreales	Nectriaceae	*Fusarium verticillioides**	Uncultured *Fusarium* clone GQ280339	393/401(98%)	Plant roots
							Fusarium verticillioides JX915250	391/401(98%)	Soils
42	1	5	Ascomycota	Helotiales	Dermateaceae	Demateaceae	Uncultured Demateaceae clone FJ553813	510/512(99%)	Forest soil in Canada
43	1	82	Basidiomycota	Atheliales	Atheliaceae	*Piloderma***	*Piloderma* sp. JQ711967	489/510(96%)	Ectomycorrhiza in Canada
44	1	4	Ascomycota	Hypocreales	Unassigned	*Acremonium***	*Acremonium* sp. EF577237	443/447(99%)	Unreported
45	1	6	Ascomycota	Chaetothyriales	Herpotrichiellaceae	Herpotrichiellaceae	*Cladophialophora chaetospira* KF359558	394/440(90%)	Plant root
46	6	262	Ascomycota	Hypocreales	Nectriaceae	*Fusarium***	*Fusarium* sp. KC748302	397/405(98%)	Compost
47	1	4	Basidiomycota	Sebacinales	Unassigned	Sebacinales	Sebacinales sp. HE814123	395/404(98%)	Root tips in China
48	1	10	Basidiomycota	Polyporales	Polyporaceae	*Trametes polyzona**	*Trametes polyzona* JN164980	400/410(98%)	Unreported
49	1	27	Ascomycota	Chaetothyriales	Herpotrichiellaceae	Herpotrichiellaceae	*Cladophialophora chaetospira* KF359558	482/526(92%)	Plant root in USA
50	1	313	Basidiomycota	Sebacinales	Sebacinaceae	*Sebacina***	Uncultured *Sebacina* mycobiont JQ420983	463/480(96%)	Plant
51	2	5	Ascomycota	Pleosporales	Pleosporaceae	*Epicoccum***	*Epicoccum* sp. JX014389	513/514(99%)	Plant leaf
52	4	131	Basidiomycota	Malasseziales	Malasseziaceae	*Malassezia restricta**	*Malassezia restricta* EU400587	445/447(99%)	Plant
53	1	3	Unasssigned	Unassigned	Unassigned	Fungus	Uncultured fungus clone KF800591	397/482(82%)	Indoor air in USA
54	1	2	Basidiomycota	Tremellales	Unassigned	*Trichosporon porosum**	*Trichosporon porosum* KF285994	404/405(99%)	Netherlands
55	1	5	Ascomycota	Helotiales	Hyaloscyphaceae	Hyaloscyphaceae	Hyaloscyphaceae sp. KF359568	357/363(98%)	Plant root in USA
56	1	17	Basidiomycota	Thelephorales	Thelephoraceae	Thelephoraceae	Uncultured Thelephoraceae clone AB769919	461/472(98%)	Ectomycorrhizal root tip
57	1	17	Ascomycota	Chaetothyriales	Herpotrichiellaceae	Herpotrichiellaceae	*Cladophialophora chaetospira* EU035405	383/406(94%)	Plant roots
58	2	68	Ascomycota	Helotiales	Unassigned	Helotiales	Uncultured Leotiomycetes clone HM230871	504/516(98%)	Mycorrhiza
							Uncultured Helotiales clone AB636433	492/516(95%)	Ectomycorrhizal root tip

OTU ID	Freq.	Reads	phylum	Order	Family	Indendification	Close GenBank match / Accession	Similarity	Originally reported habitat
59	1	16	Ascomycota	Unassigned	Unassigned	Ascomycota	Uncultured fungus clone JX987750	374/376(99%)	Soil in China
							Stromatonectria caraganae HQ112288	374/406(85%)	Unreported
60	1	8	Ascomycota	Chaetothyriales	Unassigned	Chaetothyriales	Uncultured Chaetothyriales clone KF385366	404/427(95%)	Rhizosphere soil
61	1	38	Ascomycota	Unassigned	Unassigned	Ascomycota	Ascomycota sp. JF273509	469/469(100%)	Ectomycorrhiza
62	1	5	Ascomycota	Hypocreales	Cordycipitaceae	*Engyodontium***	*Engyodontium album* HQ115665	404/423(96%)	Wall after water damage
63	1	7	Ascomycota	Unassigned	Unassigned	Ascomycota	Uncultured Ascomycota clone JQ991643	385/396(97%)	Ectomycorrhizal root tips
64	1	4	Basidiomycota	Agaricales	Tricholomataceae	Tricholomataceae	Tricholomataceae sp. EF060532	349/349(100%)	Sea water in USA (Hawaii)
65	1	15	Basidiomycota	Sebacinales	Sebacinaceae	*Sebacina***	Uncultured *Sebacina* mycobiont JQ420983	421/436(97%)	Plant
66	1	22	Zygomycota	Mortierellales	Mortierellaceae	*Mortierella humilis**	*Mortierella humilis* JF439486	394/397(99%)	Soil
67	1	8	Ascomycota	Pezizales	Pezizaceae	Pezizaceae	Pezizaceae sp. HE814085	316/317(99%)	Plant roots in China
68	5	41	Ascomycota	Eurotiales	Aspergillaceae	*Eurotium amstelodami**	*Eurotium* sp. KF367488	540/540(100%)	Untreated dringking water sources
							Eurotium amstelodami HQ728257	534/541(99%)	Pu-erh tea
69	1	16	Ascomycota	Helotiales	Hyaloscyphaceae	Hyaloscyphaceae	Hyaloscyphaceae sp. JQ272392	375/388(97%)	Plant root
70	1	5	Ascomycota	Hypocreales	Unassigned	*Acremonium tubakii**	Uncultured eukaryote clone GU942191	523/526(99%)	South China Sea
							Acremonium tubakii KC426993	466/467(99%)	Soil
71	1	2	Ascomycota	Chaetothyriales	Herpotrichiellaceae	*Cladophialophora***	*Cladophialophora chaetospira* EU035405	387/406(95%)	Soil in Germany
72	1	14	Ascomycota	Unassigned	Unassigned	Dothideomycetes	Dothideomycetes sp. JQ759518	447/447(100%)	Plant in USA
73	1	2	Unassigned	Unassigned	Unassigned	Fungus	Uncultured fungus clone JX334538	314/386(81%)	Soi
74	2	109	Ascomycota	Helotiales	Unassigned	Helotiales	Uncultured Helotiales clone JQ991730	470/471(99%)	Ectomycorrhizal root tips
75	1	19	Ascomycota	Saccharomycetales	Phaffomycetaceae	*Pichia pastoris**	*Pichia pastoris* FR839631	236/244(97%)	Unreported
76	2	77	Unassigned	Unassigned	Unassigned	Fungus	Uncultured fungus clone HQ662845	435/509(85%)	Soil in South Korea
77	1	5	Unassigned	Unassigned	Unassigned	Fungus	Uncultured fungus clone HQ436041	292/295(99%)	Axonopus compressus soil
78	7	156	Unassigned	Unassigned	Unassigned	Fungus	Uncultured fungus clone JX344420	296/386(77%)	Soil
79	2	167	Ascomycota	Helotiales	Helotiaceae	Helotiaceae	Uncultured Leotiomycetes clone HM230895	460/510(90%)	Mycorrhiza

OTU ID	Freq.	Reads	phylum	Order	Family	Indendification	Close GenBank match / Accession	Similarity	Originally reported habitat
							Uncultured Helotiaceae clone FJ440900	445/494(90%)	Plant roots in USA
80	1	25	Unassigned	Unassigned	Unassigned	Fungus	Uncultured soil fungus clone HM037647	421/433(97%)	Soil in Netherlands
81	1	4	Ascomycota	Pleosporales	Venturiaceae	*Venturia***	Uncultured *Venturia* clone FJ553146	420/433(97%)	Forest soil in Canada
82	1	78	Ascomycota	Helotiales	Hyaloscyphaceae	Hyaloscyphaceae	Uncultured Hyaloscyphaceae clone JX317338	460/467(99%)	Plant roots
83	1	2	Ascomycota	Xylariales	Amphisphaeriaceae	*Pestalotiopsis vismiae**	*Pestalotiopsis* sp. HE608797	369/369(100%)	Marine sponge
							Pestalotiopsis vismiae EF451801	366/369(99%)	Medicinal plant from Brazil
84	1	4	Ascomycota	Rhytismatales	Rhytismataceae	Rhytismataceae	Rhytismataceae sp. JQ272405	283/289(98%)	Plant root in USA
85	1	3	Basidiomycota	Agaricales	Amanitaceae	*Amanita fritillaria**	*Amanita fritillaria* JF273505	424/425(99%)	Ectomycorrhiza in China
86	1	66	Ascomycota	Helotiales	Unassigned	Helotiales	Uncultured Helotiales clone AB636433	465/466(99%)	Ectomycorrhizal root tip in China
87	5	277	Ascomycota	Hypocreales	Nectriaceae	*Fusarium solani**	*Fusarium solani* JN232143	542/545(99%)	Unreported
88	8	3005	Ascomycota	Saccharomycetales	Phaffomycetaceae	*Pichia pastoris**	*Pichia pastoris* FR839630	389/401(97%)	Unreported
89	1	4	Unassigned	Unassigned	Unassigned	Fungus	Uncultured fungus clone JX898595	346/365(95%)	Cave and mine
90	1	31	Chytridiomycota	Rhizophydiales	Unassigned	Rhizophydiales	*Betamyces americaemeridionalis* EF585664	441/515(86%)	Unreported
91	1	5	Unassigned	Unassigned	Unassigned	Fungus	Uncultured soil fungus clone JQ666673	269/338(80%)	Forest soil in China (Changbai)
92	1	33	Unassigned	Unassigned	Unassigned	Fungus	Uncultured fungus clone FN391362	399/494(81%)	Ground soil in France
93	5	138	Ascomycota	Hypocreales	Nectriaceae	*Fusarium***	Uncultured *Fusarium* clone GQ280339	379/387(98%)	Roots
94	1	26	Basidiomycota	Boletales	Boletaceae	*Boletellus obscurococcineus**	*Boletellus obscurococcineus* AB973723	348/350(99%)	Macro fungi in Japan
95	8	3321	Ascomycota	Hypocreales	Nectriaceae	*Fusarium oxysporum**	*Fusarium* sp. KF472154	547/547(100%)	Ectomycorrhiza in coastal forest
							Fusarium oxysporum KM005082	546/547(99%)	Plant in Mexico
96	1	59	Ascomycota	Helotiales	Unassigned	Helotiales	Uncultured fungus clone KF007259	508/515(99%)	Root tip in Portugal
							Helotiales sp. HE814129	448/448(100%)	Root tips in China
97	1	4	Basidiomycota	Tremellales	Unassigned	Tremellales	Tremellales sp. EF060950	366/377(97%)	Sea water in USA (Palmyra Atoll)
98	1	9	Ascomycota	Chaetosphaeriales;	Chaetosphaeriaceae	*Chloridium virescens**	Uncultured *Chaetosphaeria* clone JX317418	519/528(98%)	Plant roots in Forest
							Chloridium virescens JX684009	466/475(98%)	Decaying wood-saprophyte

OTU ID	Freq.	Reads	phylum	Order	Family	Indendification	Close GenBank match / Accession	Similarity	Originally reported habitat
99	1	7	Basidiomycota	Sebacinales	Unassigned	Sebacinales	Sebacinales sp. KF359622	345/355(97%)	Plant roots in USA
100	1	7	Basidiomycota	Sporidiobolales	Unassigned	*Curvibasidium cygneicollum**	Sporidiobolales sp. EF060827	355/367(97%)	Sea water in USA (Hawaii)
							Curvibasidium cygneicollum AF444525	325/328(99%)	Unreported
101	5	452	Unassigned	Unassigned	Unassigned	Fungus	Uncultured soil fungus clone EU480016	292/371(79%)	Semiarid grassland
102	2	3	Ascomycota	Thelebolales	Thelebolaceae	*Thelebolus globosus**	*Thelebolus globosus* JX171196	368/372(99%)	Antarctica
103	1	164	Ascomycota	Saccharomycetales	Unassigned	Saccharomycetales	*Metschnikowia pulcherrima* AY301026	378/425(89%)	Unreported
104	2	13	Basidiomycota	Atheliales	Altheliaceae	Atheliaceae	Uncultured Atheliaceae clone JQ991648	467/467(100%)	Ectomycorrhizal root tips in China
105	1	3	Ascomycota	Hypocreales	Nectriaceae	*Fusarium***	Uncultured *Fusarium* clone KF752620	232/239(97%)	Plant roots in China
106	2	1357	Unassigned	Unassigned	Unassigned	Fungus	Uncultured eukaryote clone GU941366	363/434(84%)	South China Sea
							Uncultured fungus clone AB615542	192/200(96%)	Marine subsurface sediments in Japan
107	3	32	Unassigned	Unassigned	Unassigned	Fungus	Uncultured fungus clone JX369635	288/379(76%)	Soil
108	1	695	Unassigned	Unassigned	Unassigned	Fungus	Uncultured eukaryote clone GU941366	367/440(83%)	South China Sea
							Uncultured fungus clone AB615542	189/197(96%)	Marine subsurface sediments in Japan
109	3	9	Chytridiomycota	Unassigned	Unassigned	Chytridiomycota	Uncultured Chytridiomycota clone HQ219419	333/431(77%)	Dimictic and eutrophic lake in France
110	3	448	Basidiomycota	Russulales	Unassigned	Russulales	Russulales sp. AB846966	136/158(86%)	Plant roots
111	5	48	Unassigned	Unassigned	Unassigned	Fungus	Uncultured fungus clone JX342287	296/389(76%)	Soil
							Uncultured Chytridiaceae clone EU754999	179/212(84%)	Soil and roots of oilseed rape
112	2	11	Unassigned	Unassigned	Unassigned	Fungus	Uncultured eukaryote clone KJ181484	174/179(97%)	South China Sea
113	2	4	Unassigned	Unassigned	Unassigned	Fungus	Uncultured fungus clone AB615542	174/179(97%)	Marine subsurface sediments in Japan

***species accepted (sequence identities ≥ 97%,); **genus accepted (sequence identities between 95% and 97%).**

Table S2. Distribution of the 113 OTUs found in the 8 sediment samples, including their number of reads.

OTU ID	K1	K2	K3	K4	K5	K6	K7	K8
1	2469	2289	2854	2832	12	0	7	6583
2	0	0	0	0	0	24	0	0
3	0	0	0	0	0	3	0	0
4	1075	665	931	853	3	30	11	2127
5	0	0	0	0	1	5	1	0
6	0	0	0	0	0	5	0	0
7	0	0	0	0	0	0	0	2
8	6	6	20	16	0	1	0	31
9	0	3	0	0	0	0	0	0
10	6	10	3	0	19	32	21	4
11	0	0	0	0	3	0	15	0
12	0	0	0	0	0	2	0	0
13	0	0	0	0	3	0	0	0
14	2	2	15	14	0	0	0	6
15	0	0	0	0	0	25	12	0
16	0	0	0	0	0	3	0	0
17	0	0	0	0	0	6	0	0
18	0	0	0	0	0	16	0	0
19	0	0	0	0	0	8	0	0
20	0	0	0	3	0	0	0	0
21	0	0	1	0	0	0	0	6
22	0	1	0	0	0	9	0	0
23	0	0	0	0	4	7	0	2
24	0	0	0	0	0	2	0	4
25	0	1	0	4	3	9	0	0
26	3	2	0	1	3	0	0	0
27	0	0	0	0	1	11	0	0
28	0	0	0	0	0	5	0	0
29	36	34	6	0	3	1	132	5
30	0	0	0	0	2	0	0	0
31	0	0	0	0	1	2	0	3
32	0	0	0	0	0	61	0	0
33	0	0	0	0	0	21	0	0
34	121	165	19	3	1	11	2824	3
35	0	0	0	0	0	15	0	0
36	0	0	0	0	0	3	0	0
37	0	0	0	0	0	4	0	0
38	511	333	84	7	9	17	677	7
39	0	24	0	0	3	59	1696	28
40	0	0	0	0	0	64	0	0

OTU ID	K1	K2	K3	K4	K5	K6	K7	K8
41	0	3	0	2	0	0	0	1
42	0	0	0	0	0	5	0	0
43	0	0	0	0	0	82	0	0
44	0	0	0	0	0	0	4	0
45	0	0	0	0	0	6	0	0
46	43	40	41	40	0	10	0	88
47	0	0	0	0	0	4	0	0
48	0	0	0	0	0	10	0	0
49	0	0	0	0	0	27	0	0
50	0	0	0	0	0	313	0	0
51	0	0	0	0	2	3	0	0
52	0	7	1	0	2	121	0	0
53	0	0	0	0	0	3	0	0
54	0	0	0	0	0	2	0	0
55	0	0	0	0	0	5	0	0
56	0	0	0	0	0	17	0	0
57	0	0	0	0	0	17	0	0
58	0	0	0	0	0	28	40	0
59	0	0	0	0	0	16	0	0
60	0	0	0	0	0	8	0	0
61	0	0	0	0	0	38	0	0
62	0	0	0	0	0	5	0	0
63	0	0	0	0	0	7	0	0
64	0	0	0	0	0	4	0	0
65	0	0	0	0	0	15	0	0
66	0	0	0	0	0	22	0	0
67	0	0	0	0	0	8	0	0
68	33	3	2	2	1	0	0	0
69	0	0	0	0	0	16	0	0
70	0	0	0	0	5	0	0	0
71	0	0	0	0	0	2	0	0
72	0	0	0	0	0	14	0	0
73	2	0	0	0	0	0	0	0
74	0	4	0	0	0	105	0	0
75	0	0	0	0	0	0	19	0
76	0	0	0	0	0	65	12	0
77	0	0	0	0	0	5	0	0
78	11	45	0	4	36	25	30	5
79	0	0	0	0	0	55	112	0
80	0	0	0	0	0	25	0	0
81	0	0	0	0	0	4	0	0
82	0	0	0	0	0	78	0	0
83	0	0	0	0	0	2	0	0

OTU ID	K1	K2	K3	K4	K5	K6	K7	K8
84	0	0	0	0	0	4	0	0
85	0	0	0	0	0	3	0	0
86	0	0	0	0	0	66	0	0
87	44	51	34	25	0	0	0	123
88	526	371	91	8	6	27	1970	6
89	0	0	0	0	0	4	0	0
90	0	0	0	0	0	31	0	0
91	0	0	0	0	5	0	0	0
92	0	0	0	0	0	33	0	0
93	17	23	12	16	0	0	0	70
94	0	0	0	0	0	26	0	0
95	610	405	631	566	4	1	5	1099
96	0	0	0	0	0	59	0	0
97	0	0	0	0	0	0	4	0
98	0	0	0	0	0	9	0	0
99	0	0	0	0	0	7	0	0
100	0	0	0	0	0	7	0	0
101	1	13	0	0	0	120	314	4
102	0	0	0	0	0	1	2	0
103	0	0	0	0	0	0	164	0
104	0	0	0	0	0	3	10	0
105	0	0	0	0	0	0	0	3
106	0	1	0	0	1356	0	0	0
107	0	1	0	0	0	6	25	0
108	0	0	0	0	695	0	0	0
109	3	1	0	0	5	0	0	0
110	0	0	1	0	6	440	0	1
111	7	1	0	1	32	0	0	7
112	0	0	0	0	2	9	0	0
113	1	3	0	0	0	0	0	0

Table S3. Dissimilarity test (multiple response permutation procedure, MRPP) for significant differences in fungal community composition among the 3 basins using QIIME 1.8.0 software.

	group A	groupB	groupC	groupA vs. groupB vs. groupC	groupA vs. groupB	groupA vs. groupC	groupB vs. groupC
delta	0.1324	0.2782	0.9557	-	-	-	-
n#	2	3	3	-	-	-	-
A-value	-	-	-	0.3048	0.1921	0.2658	0.2626
Observed delta	-	-	-	0.4958	0.2199	0.6263	0.617
Expected delta	-	-	-	0.7132	0.2722	0.8531	0.8367
Significance of delta	-	-	-	0.005	0.086	0.086	0.107

group A: K1, and K2

group B: K3, K4, and K8

group C: K5, K6, and K7

Number of samples in each group

Based on 999 permutations

Table S4. Primers used for 454 pyrosequencing of the 8 sediment samples.

Sample ID	Forward Primer (5'-adaptor A-barcode-ITS1F-3')	Reverse Primer (5'-adaptor B-ITS4-3')
K1	CCATCTCATCCCTGCGTGTCTCCGACGACT- AGCAGATG -CTTGGTCATTTAGAGGAAGTAA	CCTATCCCCTGTGTGCCTTGGCAGTCGACT-TCCTCCGCTT ATTGATATGC
K2	CCATCTCATCCCTGCGTGTCTCCGACGACT - AGCTACGT -CTTGGTCATTTAGAGGAAGTAA	CCTATCCCCTGTGTGCCTTGGCAGTCGACT-TCCTCCGCTT ATTGATATGC
K3	CCATCTCATCCCTGCGTGTCTCCGACGACT - AGACACAG -CTTGGTCATTTAGAGGAAGTAA	CCTATCCCCTGTGTGCCTTGGCAGTCGACT-TCCTCCGCTT ATTGATATGC
K4	CCATCTCATCCCTGCGTGTCTCCGACGACT - ACTGCTCT -CTTGGTCATTTAGAGGAAGTAA	CCTATCCCCTGTGTGCCTTGGCAGTCGACT-TCCTCCGCTT ATTGATATGC
K5	CCATCTCATCCCTGCGTGTCTCCGACGACT - AGCAGATG -CTTGGTCATTTAGAGGAAGTAA	CCTATCCCCTGTGTGCCTTGGCAGTCGACT-TCCTCCGCTT ATTGATATGC
K6	CCATCTCATCCCTGCGTGTCTCCGACGACT - ACACACTG -CTTGGTCATTTAGAGGAAGTAA	CCTATCCCCTGTGTGCCTTGGCAGTCGACT-TCCTCCGCTT ATTGATATGC
K7	CCATCTCATCCCTGCGTGTCTCCGACGACT – ACACGTCA -CTTGGTCATTTAGAGGAAGTAA	CCTATCCCCTGTGTGCCTTGGCAGTCGACT-TCCTCCGCTT ATTGATATGC
K8	CCATCTCATCCCTGCGTGTCTCCGACGACT – AGTCGTCA -CTTGGTCATTTAGAGGAAGTAA	CCTATCCCCTGTGTGCCTTGGCAGTCGACT-TCCTCCGCTT ATTGATATGC

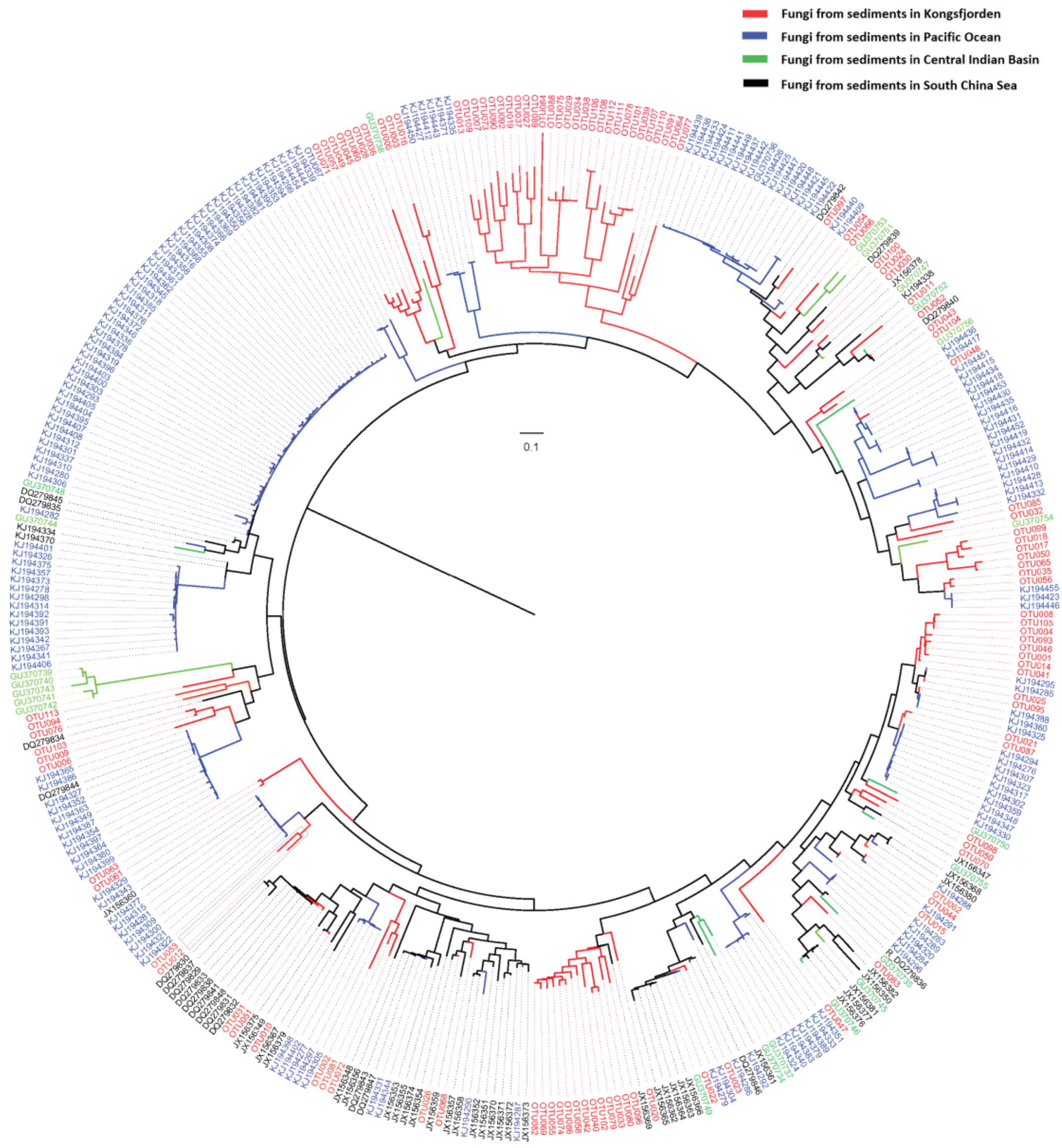

Figure S1. A phylogenetic tree showing the relationships between the sedimentary fungi in Kongsfjorden (113 representative OTUs) and fungi in sediments from other regions, including Pacific Ocean (NCBI Accession Nos. KJ194276 to KJ194455), South China Sea (NCBI Accession Nos. JX156347 to JX156382 and DQ279829 to DQ279848) and Central Indian Basin (NCBI Accession Nos. GU370734 to GU370756). The phylogenetic tree was constructed based on the ITS sequence using the Neighbor-Joining method with the maximum composite likelihood substitution model (MEGA v. 6.0 software).

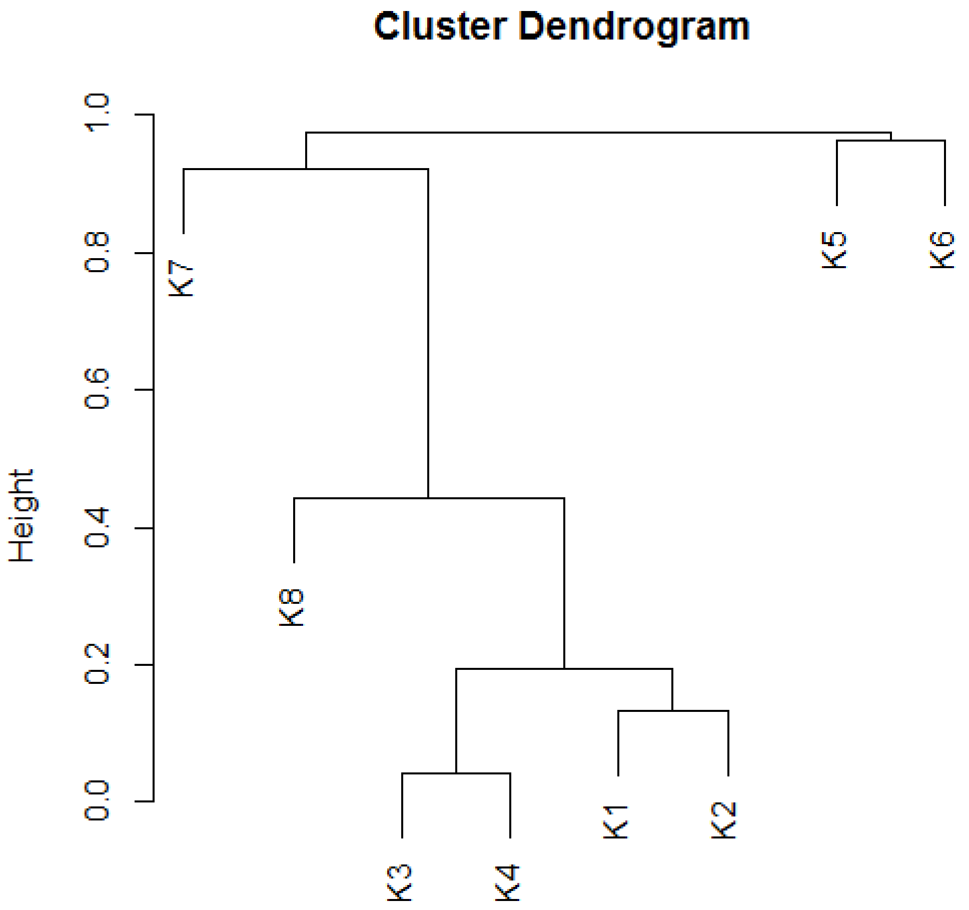

Figure S2. Hierarchical clustering analysis of fungal communities in the 8 sediment samples based Bray-Curis-transformed abundance data for OTUs using R 3.1.1 statistical software.
